# INCONSISTENT ESTIMATES OF POOR HEALTH BETWEEN EUROPEAN SURVEYS—A MATTER OF SURVEY DESIGN?

**DOI:** 10.1093/geroni/igad104.1737

**Published:** 2023-12-21

**Authors:** Susanne Kelfve, Linda Enroth

**Affiliations:** Linköping University, Norrköping, Ostergotlands Lan, Sweden; Tampere University, Tampere, Pirkanmaa, Finland

## Abstract

Several studies have reported about inconsistencies in survey estimates on health, both within and between countries. One potential source of inconsistency in results from population surveys is survey quality. In particular, the prevalence of poor health among older people is sensitive for survey design factors and the inclusion of older people with poor health. We aimed to explore the association between survey design factors and within-country differences (between EU-SILC and SHARE) in estimated prevalence of poor health (long-term illness and global activity limitation (GALI)) among older people. The analyses include people 65 years and older from 25 European countries with data collections both in SHARE and EU-SILC 2017. Results show substantial differences in sociodemographic factors and estimates of poor health, both between and within countries. The within-country differences in the prevalence of long-term illness ranged from -18 percentage points for men in Denmark (lower in EU-SILC compared with SHARE) to 42 percentage points for women in Cyprus (higher in EU-SILC compared with SHARE). The prevalence of GALI showed similar patterns. We conclude that estimates of poor health, measured by long-term illness and GALI, have great variation between surveys within the same country. Survey design factors, such as response rate and the type and number of survey mode seems to explain part of these inconsistencies. Population surveys are widely used for monitoring health and the results from these surveys are utilized in health policy planning. Thus, it is crucial to understand the possible impact of survey design on health estimates.

